# Severe Cardiomegaly in Congestive Heart Failure: Postmortem Evidence of Cardiac Remodeling

**DOI:** 10.1016/j.jaccas.2025.105822

**Published:** 2025-11-26

**Authors:** Ty R. Mote, Christopher T. Scroggins, Alexis Gillett, Caitlin Yoakum

**Affiliations:** a Arkansas College of Osteopathic Medicine, Fort Smith, Arkansas, USA; b Alice L. Walton School of Medicine, Bentonville, Arkansas, USA

**Keywords:** atrial fibrillation, cardiomyopathy, chronic heart failure, hypertension

## Abstract

**Background:**

Cardiomegaly is the gross manifestation of advanced cardiac remodeling, a compensatory response to prolonged hemodynamic stress. Direct postmortem examination of cardiac remodeling is rare, because cardiomegaly is clinically identified through radiologic imaging.

**Case Summary:**

An 81-year-old male cadaveric donor with congestive heart failure as the known cause of death was dissected under standard procedure. Gross examination revealed severe enlargement of the heart and marked dilation of the aorta. Microscopic examination demonstrated significant left ventricular myocyte hypertrophy. Comparative analysis with 5 additional male donors confirmed that the structural findings were statistically significant across all measurements.

**Discussion:**

The known history, gross structural findings, and microscopic features favor a hypertensive etiology and reinforce the structural basis of heart failure usually inferred from clinical imaging.

**Take-Home Message:**

Postmortem visualization of cardiac structural morphology provides a unique educational opportunity to correlate gross pathology with common etiologies and appreciate how cardiovascular remodeling contributes to the clinical progression of congestive heart failure.

## Graphical abstract

**Figure:**
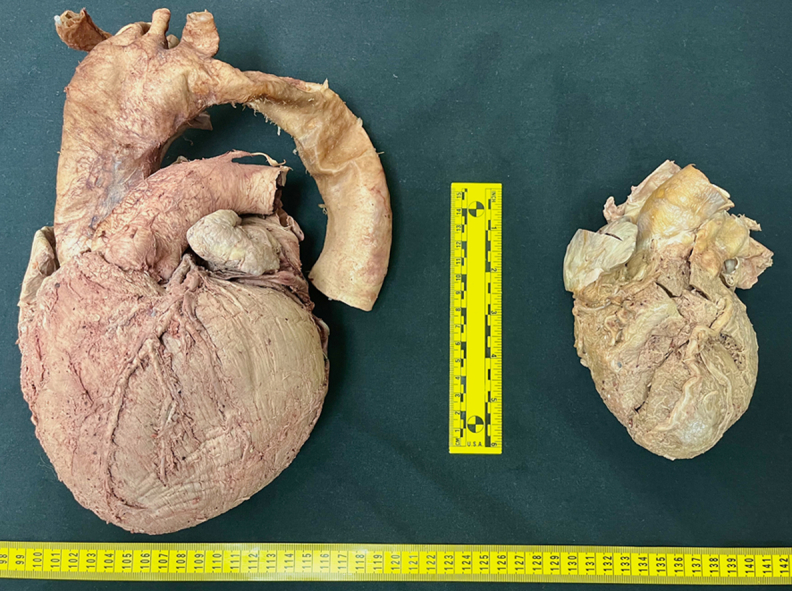

## History of Presentation

An 81-year-old male cadaveric donor with congestive heart failure as the known cause of death, and an additional longstanding medical history of persistent atrial fibrillation, was dissected under standard procedure in a graduate-level health care professional course. The donor was measured at a height of 186 cm with an unknown weight. The body mass index was not documented, but visual estimation suggested class II obesity.

**Take-Home Messages**

• This case highlights the educational value of cadaveric dissection in clinical training by providing direct visualization of cardiac remodeling associated with the advanced stages of heart failure, which would otherwise be viewed clinically by radiologic imaging modalities.
• This unique perspective reinforces key pathophysiologic principles in the progression of cardiomyopathy, emphasizing the importance of clinical awareness and decision-making for preventing irreversible cardiac remodeling.

**Figure 1:**
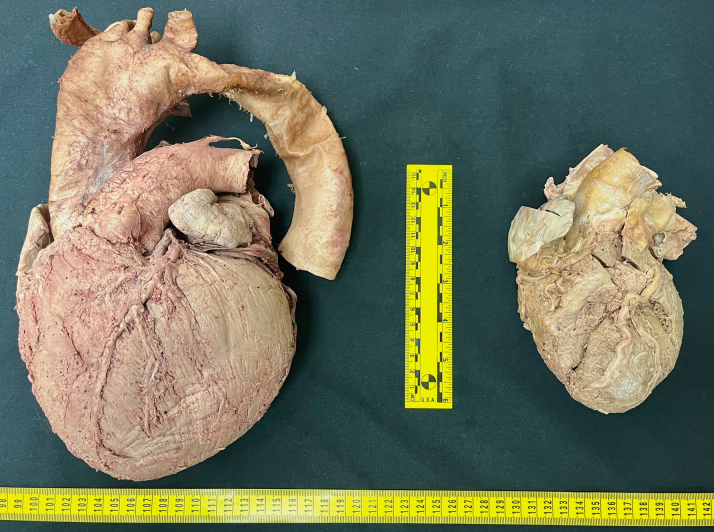
Heart Comparison Gross comparison between the case heart (left) and the heart of another cadaveric donor (right) in the same cohort after dissection of the thoracic cavity.

**Figure 2:**
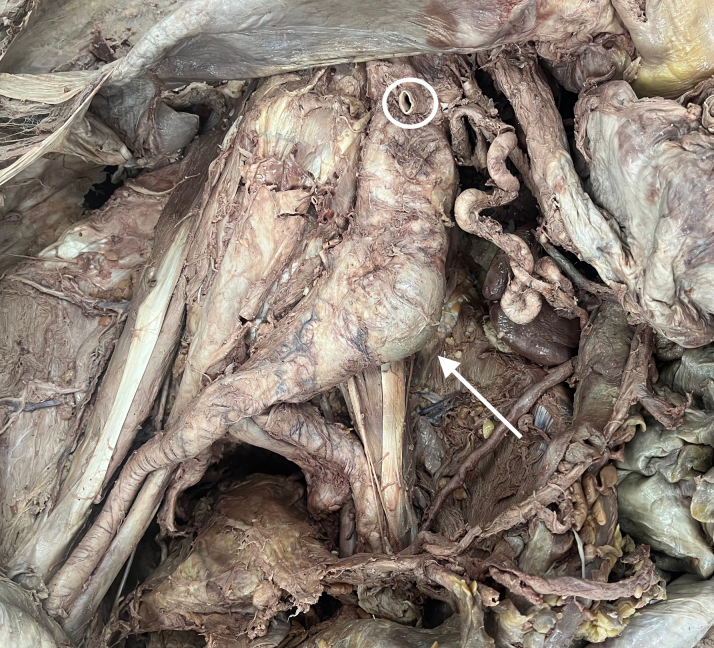
Abdominal Cavity Post-Dissection Aneurysmal dilation of the abdominal aorta (arrow), measured at 7.58 cm in length and 5.62 cm in width, inferior to the superior mesenteric artery (circle).

On opening the anterior thoracic cavity, gross examination revealed structural asymmetry of the thoracic cage, severe enlargement of the heart (Figure 1), marked dilation of the ascending aorta, and reduced left lung size. A cardiac resynchronization device was also located with endocardial attachments in the right ventricle, right atrium, and right coronary sinus. Observations with further dissection into the abdominal cavity included bilateral renal cysts, anomalous renal vasculature, and aneurysmal dilation of the abdominal aorta (Figure 2).

**Figure 3:**
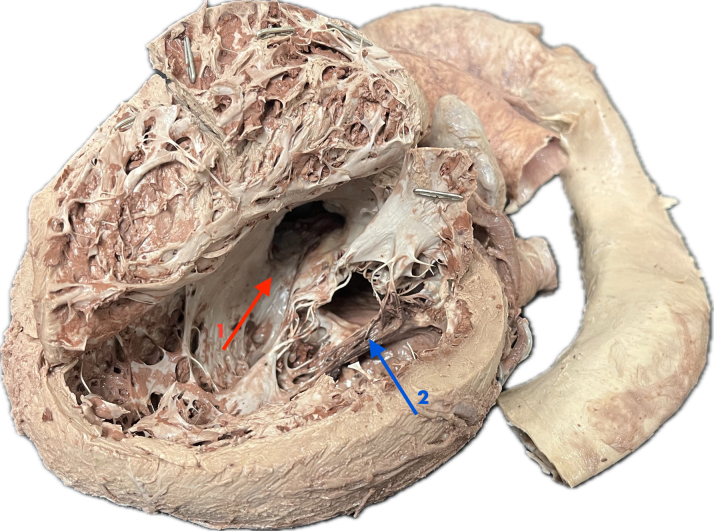
Gross Appearance of the Left Ventricular Chamber The case heart after removal of the anterior wall, with visualization of the dilated aortic (red arrow 1) and mitral (blue arrow 2) orifices. The left ventricle is markedly dilated, and the chamber walls are thin.

After removal from the thoracic cavity, a detailed dissection was performed on the structural components of the heart (Figure 3). The coronary arteries showed no significant atherosclerotic features, and the myocardium appeared uniformly brown, without mottling or scarring. The left heart chambers appeared grossly dilated with abnormally thin walls. Examination of the cardiac valves revealed circumferential dilation at the aortic, mitral, and pulmonic orifices with age-related degeneration of the valve leaflets.

## Differential Diagnoses

Although the cause of death was determined to be congestive heart failure, any underlying pathology, in addition to atrial fibrillation, that could have potentially contributed to the stark multisystem structural morphology remained in question. Given the high likelihood of a chronic disease course in this case, certain genetic variants, physiologic factors, metabolic disorders, and environmental exposures were considered. Continued evaluation of the heart through histologic analysis and molecular genetic studies was pursued.

## Investigations

Microscopic examination of the left ventricle revealed severe myocyte hypertrophy with abundant subendocardial and transmural interstitial fibrosis. Histologic analysis of the ascending aorta demonstrated moderate degenerative changes, including elastic fiber fragmentation, extensive intralamellar and translamellar mucoid extracellular matrix accumulation, and laminar medial necrosis. No evidence of myocarditis, amyloidosis, hemochromatosis, or an underlying storage disorder was identified. In addition, molecular genetic studies were unable to identify any pathogenic gene variant, based on current published data, that contributed to the structural morphology present in this case.

A quantitative comparison was also conducted between the structural findings from this dissection and the mean of those from 5 additional male cadaveric donors in the same cohort. Of note, the cardiac mass was measured at 810 g (cohort mean = 348 g), and a modified cardiothoracic ratio, determined as cardiac width divided by thoracic cavity width, was calculated at 58.4%. Additional measurements of the heart demonstrated significant deviation in this case from what would be expected for a male donor at the time of death (Table 1).

**Table 1:** Quantitative Comparison of Cardiovascular Measurements Among the Cadaveric Cohort

Variable Measured (cm)	Case Heart	Cohort Heart 1	Cohort Heart 2	Cohort Heart 3	Cohort Heart 4	Cohort Heart 5
Length, apex to base	16.83	12.67	12.54	12.01	13.17	15.66
Transverse diameter^a^	16.13	10.85	9.06	9.95	12.22	12.63
Ascending aorta diameter^b^	5.87	4.26	3.59	3.25	2.68	3.51
Aortic arch diameter^c^	4.36	2.21	2.56	2.56	2.32	3.49
Thoracic aorta diameter^d^	2.94	2.32	2.17	2.03	2.16	2.08
Abdominal aorta diameter^e^	4.39	2.97	2.31	2.21	2.14	2.58

a Measured at the level of the atrioventricular sulcus.

b Measured superior to the left auricle at the level of the pulmonary artery bifurcation.

c Measured between branch points of the brachiocephalic trunk and left common carotid artery.

d Measured superior to the level of the thoracoabdominal diaphragm.

e Measured at the level of the superior mesenteric artery.

Using a least-squares means analysis, the case heart had an estimated average measurement of 8.42 U compared with 5.78 U for the other 5 hearts in the cohort. The average difference was 2.64 U, which is highly statistically significant (*P* < 0.00001), indicating that the case heart was consistently and substantially larger than the other hearts in the cohort across all measurement types and unlikely due to chance.

## Discussion

The course of cardiac remodeling is dependent on the nature and duration of underlying pathology, commonly pressure or volume overload.^1^ Initial modifications to cardiomyocytes occur as an adaptive mechanism to reduce the cardiac workload.^2^ These changes are accompanied by constitutive activation of the renin-angiotensin-aldosterone system, sustained increases in sympathetic tone, and release of natriuretic peptides.^3^ Over time, this adaptive mechanism undergoes an irreversible maladaptive transformation when excessive hypertrophy, fibrosis, and contractile dysfunction occur because of continued exposure to the pathologic stimulus, culminating in heart failure.^2^ The onset of irreversible cardiac remodeling typically leads to a rapid decline, so extreme structural morphology, as seen in this case, is not routinely observed clinically and direct visualization is rare.

Dilated cardiomyopathy (DCM), a common cause of cardiomegaly resulting from irreversible remodeling, is classified by the presence or absence of ischemia. Ischemic DCM is most prevalent and often results from coronary artery disease, which was minimally evident in this case. Nonischemic DCM is complex, arising from a myriad of genetic, environmental, or physiologic factors, and is characterized as idiopathic or secondary to underlying comorbidities.^4^ Among these, nonischemic DCM secondary to hypertensive heart disease has been highly associated with the development of structural and functional cardiac abnormalities and an increased incidence of progression to congestive heart failure.^5^, ^6^, ^7^

The lack of information on this donor does present significant limitations for determining the primary factors that contributed to the severity of structural remodeling observed. The known history, gross findings, and microscopic analysis do, however, provide evidence-based characteristics that favor nonischemic DCM resulting secondary to a hypertensive etiology.

Left ventricular hypertrophy resulting from hypertension is highly associated with atrial fibrillation, a key aspect of the known history in this case.^8^ In addition, the histologic patterns and severe enlargement of the heart, specifically in the left cardiac chambers, are consistent with what would be expected in persistent atrial fibrillation secondary to chronic hypertension. Conventional knowledge suggests that cardiac remodeling of the left ventricle due to hypertension typically leads to concentric hypertrophy. Interestingly, a recent review indicates that eccentric hypertrophy is the more prevalent outcome in advanced disease.^9^ The maladaptive structural changes of the heart in chronic hypertension and persistent atrial fibrillation synergistically accelerate the progression of irreversible cardiac remodeling and the clinical manifestation of decompensated heart failure.

The aneurysmal dilation and histologic characteristics of the aorta are further evidence that hypertension might have been a primary contributor to the findings in this case. Systemic hypertension is known to significantly affect vascular functionality, with elevated central pressure being particularly detrimental to large arteries. Prolonged hemodynamic stress in the great vessels results in a similar remodeling process, as seen in the heart, that decreases vascular compliance and elasticity, with progressive stiffening and dilation. These changes are exacerbated by normal vascular deterioration associated with aging.^10^

Structural enlargement of the heart alone does not provide diagnostic clarity, but its clinical significance is heavily implicated in disease prognosis, as it commonly presents alongside symptoms indicative of congestive heart failure.^1^ Regardless of the underlying etiology that led to such severe structural manifestations, this case informs clinical decision-making by underscoring the importance of early recognition and aggressive management of risk factors for preventing irreversible cardiovascular damage and increasing positive patient outcomes.

## Conclusions

The objective of this dissection was not to formulate a postmortem diagnosis, but rather to consider the structural findings from a clinical perspective by investigating the presence of underlying pathology to achieve a greater understanding for how this case may have progressed. Cadaveric dissection has significant educational value in demonstrating the structural morphologic changes associated with chronic cardiovascular disease. In contrast with radiologic imaging or other diagnostic methods, the alternative perspective in this case provides a unique opportunity to directly connect the gross structural features of cardiac remodeling to the clinical manifestation of congestive heart failure and consider the importance of patient-centered care for modifying cardiovascular risk factors.

## Funding Support and Author Disclosures

Genetic testing for this case report was supported by institutional funds from Alice L. Walton School of Medicine in Bentonville, Arkansas. The authors have reported that they have no relationships relevant to the contents of this paper to disclose.
